# Comprehensive Unified Regimen for Eliminating Undiagnosed/Untreated Aortic Valve Stenosis: Algorithm Validation for Identifying Aortic Stenosis and Treatment Disparities

**DOI:** 10.1016/j.shj.2025.100755

**Published:** 2025-11-05

**Authors:** Daniel Mitchell, Dhairya Patel, Jesse Navarrette, Raj Makkar, Susan Cheng, Joseph E. Ebinger

**Affiliations:** Department of Cardiology, Smidt Heart Institute, Cedars-Sinai Medical Center, Los Angeles, California, USA

**Keywords:** Aortic stenosis, Electronic health record, Social determinants of health, Surgical aortic valve replacement, Transcatheter aortic valve replacement

## Abstract

**Background:**

Severe aortic stenosis (sAS) leads to high morbidity and mortality when left untreated. We sought to develop and validate an algorithm-based rules engine to identify patients with untreated sAS and to evaluate differences between those who did and did not subsequently receive guideline-concordant treatment with aortic valve replacement (AVR).

**Methods:**

We curated discrete and nondiscrete data from our echocardiography system, then created a rules engine to identify and grade aortic stenosis. We assessed sensitivity and specificity of the rules engine to identify sAS using manual adjudication. We additionally conducted a retrospective cohort analysis to identify demographic and socioeconomic factors associated with receipt of guideline-concordant AVR treatment for sAS.

**Results:**

The rules engine demonstrated 100% sensitivity and 95.4% specificity for identifying sAS across n ​= ​2162 echocardiographic studies from unique patients. Univariate analyses revealed patients with untreated sAS were more likely to be older and female, with no appreciated differences by race, ethnicity, insurance status, or neighborhood-level socioeconomic scores. In multivariable analyses, older individuals, women, and those with Medicare/Medicare advantage were less likely to undergo AVR. Among treated patients, those who underwent surgical AVR were more likely to be younger, male, and have lower socioeconomic neighborhood scores.

**Conclusions:**

Untreated sAS is prevalent but can be accurately identified at scale using an echocardiogram report-based rules engine. Disparities in the receipt of AVR persist, particularly among women, older adults, and patients with nonprivate insurance coverage. The systematic use of automated algorithmic protocols may facilitate valvular heart disease identification and reduction of treatment disparities.

## HIGHLIGHTS

• Development and validation of an echocardiogram-based rules engine identified a high prevalence (11%) of untreated severe aortic stenosis.
• The developed rules engine demonstrated high sensitivity (100%) and specificity (95%) for categorizing severe aortic stenosis.
• Disparities in the receipt of guideline-concordant aortic valve replacement persist, particularly among women, older adults, and patients with nonprivate insurance coverage.

## Introduction

Aortic stenosis (AS) remains among the most morbid and mortal forms of valvular heart disease, with a prevalence that is estimated to double by the year 2050.^1^ Despite this rising prevalence, delayed treatment remains common. Less than half of patients with moderate-to-severe AS and fewer than two-thirds of patients with severe AS receive guideline-recommended treatment with aortic valve replacement (AVR) over a 4-year period, during which mortality among untreated patients approaches 50%.^2^ These findings underscore the need for novel strategies to improve identification of AS at the population level and enhance our understanding of barriers to care.

Advances in machine learning and algorithmic models have the potential to revolutionize the recognition of valvular heart disease. Prior work has demonstrated feasibility of developing validated machine learning algorithms capable of identifying AS from echocardiographic images, predict the course of AS progression, augment AS grading, and predict mortality following transcatheter aortic valve replacement (TAVR).^3^, ^4^, ^5^, ^6^ Despite these advances, clinical implementation of algorithms designed to screen for and guide management of untreated AS at the health system level remain elusive. Specifically, there is a lack of streamlined, generalizable solutions to help overcome siloed care in which AS identified by echocardiography fails to result in timely clinical diagnosis and management. Thus, motivated by the opportunity to reduce the morbidity and mortality of untreated AS, we sought to create and validate a novel rules engine designed to identify AS at the population level as well as analyze disparities in treatment patterns to aid in addressing existing barriers to care.

## Materials and Methods

### Clinical Rules Engine Development

This study was performed at Cedars-Sinai Medical Center, a large integrated health care system in Los Angeles. An automated rules engine was developed to identify and classify patients with AS based on clinically generated data. Relevant clinical and echocardiographic data were mapped from our institutional electronic health record (Epic, Verona, WI) and echocardiography system (Syngo; Siemens, Malvern, PA). From the electronic health record (EHR), we extracted basic demographics (including sex, defined as “legal sex” in the EHR), patient census tract (for mapping to neighborhood-level social determinants of health [SDoH]), and insurance status. From the echocardiography system, we extracted quantitative (mean gradient, peak velocity, calculated aortic valve area) and qualitative (echocardiographer interpretation) measures from transthoracic echocardiograms. Echocardiograms with prosthetic aortic valves were excluded.

The criteria used to identify AS followed a hierarchical approach, first using natural language processing to perform a qualitative analysis of echocardiogram reports to determine any mention of degree of AS, leaflet restriction, or comment on abnormal gradients. In cases where no qualitative text was found, quantitative measurements were evaluated using raw data extracted from the Siemens system in which the measurements were made. Quantitatively, aortic valve area was first analyzed, and in cases without aortic valve area recorded measurements, mean gradients were evaluated. Quantitative values were used to grade AS in accordance with 2020 American College of Cardiology/American Heart Association Valvular Heart Disease Guidelines.^7^ If quantitative and qualitative classifications differed, the written interpretation by the reading board-certified echocardiography attending was considered the true AS severity class. Model output was displayed using Tableau (Tableau Software LLC, Mountain View, CA) and was updated weekly.

### Rules Engine Validation

We performed a retrospective observational cohort analysis of all transthoracic echocardiograms completed during calendar year 2023. We assessed the accuracy of AS identification by manual adjudication of a convenience sample of 200 randomly selected echocardiograms identified by the rules engine as having untreated moderate to severe (sAS) or worse AS, 200 randomly selected echocardiograms identified as having moderate or less severe AS, and 200 randomly selected echocardiograms not identified by the rules engine as having any degree of AS. Manual adjudication occurred in June 2024 and patients were confirmed to have untreated sAS if they had not undergone AVR by June 10, 2024. Patients whose echocardiogram was randomly selected for manual review that previously declined to participate in chart review (i.e., those who previously opted out of research studies when receiving care) were omitted. Rules engine classification and manual adjudication were considered concordant based on the agreement of AS severity as either moderate or less or as moderate/severe or worse. In secondary analyses, classifications were considered concordant if within half a grading scale (i.e., moderate and moderate/severe were considered concordant, whereas moderate and severe were considered nonconcordant). Multivariable logistic regression was performed to evaluate for factors associated with rules engine misclassification of AS severity.

### Prevalence and Correlates of Differences in AS Treatment Status

Following development and validation of the rules engine, we applied the rules engine to identify the prevalence of patients with sAS. We then examined for the presence of sociodemographic factors (e.g., SDoH variables detailed below) that may differ among patients with sAS who did versus did not go on to receive guidelines concordant treatment with AVR. Specifically, patients were classified by treatment status: if they underwent AVR (either surgical aortic valve replacement [SAVR] or TAVR) or did not by June 10, 2024. To ensure cohort completeness, we additionally included participants who underwent AVR in 2023 but whose most recent echocardiogram was performed at the end of 2022 (n = ​80). We performed multivariable logistic regression, controlling for age, sex, race/ethnicity, insurance status, and neighborhood level SDoH to identify factors associated with receiving AVR. We secondarily assessed for differences in modality of treatment (SAVR vs. TAVR) among those who did receive AVR for sAS.

### Social Determinants of Health Variables

Neighborhood-level SDoH data were obtained by linking patient census tracts’ to the Public Health Alliance of Southern California database, which includes information on neighborhood socioeconomic and demographic factors at the census-tract level.^8^ This database incorporates publicly available sociodemographic data, including from the American Census Survey, with community level variables such as tree density, air quality and pollution measures, and percentage of registered voters.^8^ A total of 23 weighted variables across 8 domains (economic, education, social, transportation, health care access, neighborhood, housing, and clean environment) are used to calculate the Healthy Places Index (HPI; version 3.0).^8^ We used the HPI Score as a marker of neighborhood-level SDoH, with higher scores corresponding to more favorable SDoH.

### Statistical Analyses

Descriptive statistics are presented for patient demographics, insurance status, and SDoH for patients identified as having any form of AS by the rules engine in 2023, with subsequent multivariable logistic regression used to evaluate factors associated with AS severity misclassification. Next, we used univariate and multivariable-adjusted logistic regression to examine the association of demographics, insurance status, and SDoH characteristics with AS treatment status, comparing both untreated patients with treated patients. In secondary analyses, we repeated this process to evaluate for differences by treatment modality (TAVR vs. SAVR). In sensitivity analyses, we repeated these analyses following stratification by availability of patient SDoH data. All statistical analyses were conducted using R (v4.2.1) and considered a 2-tailed *p* ​< ​0.05 statistically significant. Institutional review board approval was obtained from Cedars-Sinai Medical Center with a waiver for informed consent.

## Results

**Figure 1:**
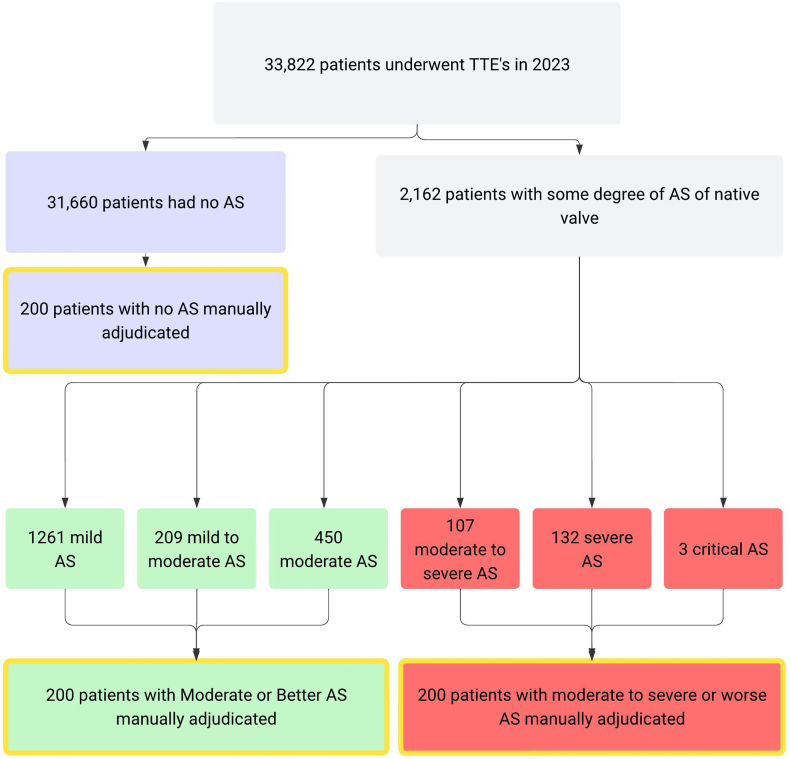
**Study selection diagram**. Abbreviations: AS: aortic stenosis; TTE, transthoracic echocardiogram.

The rules engine identified 2162 patients with sufficient data to quantify AS of any severity based on echocardiography performed during the 2023 calendar year (Figure 1). Of these, 242 (11.2%) were classified as having untreated sAS. SDoH data were missing for 326 patients (45%) due to either missing census tract or primary census tract outside of the state of California (Supplemental Table 1).

Manual adjudication revealed 10 (5%) misclassified cases. In all 10 cases, the rules engine overestimated AS severity (i.e., classified an echocardiogram as having sAS, whereas manual adjudication classified the AS as moderate or less in severity), with 2 of these patients ultimately having no AS on manual review (Table 1). Additional manual adjudication of 200 randomly selected echocardiograms from our echocardiography database (i.e., all transthoracic echocardiograms performed in 2023) did not reveal any patients with sAS that were missed by the rules engine. This resulted in the rules engine having a sensitivity of 100% and specificity of 95.4% for identifying sAS. Multivariable logistic regression did not reveal any patient-related factors associated with misclassification of AS severity (Supplemental Figure 1).

**Table 1:** Characteristics of patients identified as having at least some degree of aortic stenosis by the rules engine

	Moderate or less (n ​= ​210)	Mod/severe or worse (n ​= ​190)	All patients (n ​= ​2162)
Age, mean ± SD	77.23 ± 12.86	80.86 ± 11.92	77.77 ± 12.7
Sex, n (%)			
Male	118 (56.2%)	99 (52.1%)	1167 (53.9%)
Female	92 (43.8%)	90 (47.4%)	993 (45.9%)
Other	0 (0.0%)	1 (0.5%)	2 (0.09%)
Race, n (%)			
American Indian or Alaska Native	3 (1.4%)	0 (0.0%)	7 (0.32%)
Asian	18 (8.6%)	17 (18.9%)	206 (9.52%)
Black	22 (10.5%)	13 (6.8%)	218 (10.1%)
White	143 (68.1%)	133 (70.0%)	1450 (67.1%)
Unknown/patient declined	24 (11.4%)	27 (14.2%)	276 (12.8%)
Ethnicity, n (%)			
Hispanic	37 (17.6%)	25 (13.2%)	306 (14.2%)
Non-Hispanic	168 (80.0%)	162 (85.3%)	1797 (83.1%)
Unknown	5 (2.4%)	3 (1.6%)	59 (2.72%)
LVEF %, mean ± SD	60.1% ± 10.4	57.8% ± 13.8	57.5% ± 11.7
Grading of aortic stenosis by rules engine, n (%)			
Mild	128 (61.0%)	0 (0.0%)	1261 (58.3%)
Mild to moderate	21 (10.0%)	0 (0.0%)	209 (9.67%)
Moderate	51 (24.3%)	0 (0.0%)	450 (20.8%)
Moderate to severe	0 (0.0%)	87 (45.8%)	107 (4.95%)
Severe	8 (3.8%)	103 (54.2%)	132 (6.11%)
Critical	2 (1.0%)	0 (0.0%)	3 (0.14%)
Grading of aortic stenosis by manual adjudication, n (%)			
None	2 (1.0%)	0 (0.0%)	
Mild	86 (41.0%)	0 (0.0%)	
Mild to moderate	21 (10.0%)	0 (0.0%)	
Moderate	41 (19.5%)	0 (0.0%)	
Moderate to severe	0 (0.0%)	87 (45.8%)	
Severe	0 (0.0%)	103 (54.2%)	
Not specified	60 (28.6%)	0 (0.0%)	

Abbreviation: LVEF, left ventricular ejection fraction.

Next, we evaluated for characteristics associated with nontreatment of sAS. The median follow-up time from echocardiogram to end of follow-up for patients with untreated sAS was 11 months and 2 days. By comparison, the median time from echocardiogram to treatment for identified patients who underwent AVR was 3 days. In univariate analysis, compared to those who received AVR, patients with untreated sAS were generally older (76.6 ​± ​12.2 years vs. 81.6 ​± ​11.9; *p* ​< ​0.001) and more frequently female (32.2 vs. 45.9%; *p* ​< ​0.001), without significant differences by race, ethnicity, or HPI score (Table 2). In secondary analyses, compared to those treated with TAVR, those who underwent SAVR were younger (mean age 79.6 ​± ​9.9 years vs. 61.0 ​± ​11.2 years; *p* ​< ​0.001), less frequently female (34.8 vs. 18.8%; *p* ​= ​0.003), and had significantly lower mean HPI scores (0.32 ​± ​0.46 vs. 0.17 ​± ​0.55; *p* ​= ​0.048), indicative of more adverse neighborhood-level SDoH (Table 3).

**Table 2:** Univariate analysis comparing patients identified by rules engine to have untreated severe aortic stenosis. with treated patients in 2023.

	Received AVR	Untreated sAS	*p* value
N	584	242	
Age, mean ± SD	76.56 ± 12.23	81.63 ± 11.91	<0.001
Sex, n (%)			<0.001
Female	188 (32.2)	111 (45.9)	
Male	396 (67.8)	130 (53.7)	
Unknown	0 (0.0)	1 (0.4)	
Race, n (%)			0.449
White	439 (75.2)	169 (69.8)	
Asian	43 (7.4)	20 (8.3)	
Black	26 (4.5)	16 (6.6)	
Native Hawaiian or Pacific Islander	2 (0.3)	0 (0.0)	
American Indian or Alaska Native	3 (0.5)	0 (0.0)	
Other	54 (9.2)	28 (11.6)	
Unknown	17 (2.9)	9 (3.7)	
Ethnicity, n (%)			0.193
Non-Hispanic	507 (86.8)	201 (83.1)	
Hispanic	57 (9.8)	34 (14.0)	
Unknown	20 (3.4)	7 (2.9)	
Insurance, n (%)			<0.001
Private	297 (50.9)	76 (31.4)	
Medicare	218 (37.3)	139 (57.4)	
HMO	7 (1.2)	5 (2.1)	
Medicare Advantage	33 (5.7)	3 (1.2)	
Medicaid	11 (1.9)	3 (1.2)	
Unknown	18 (3.1)	16 (6.6)	
SDoH			
HPI, mean ± SD	0.30 ± 0.47	0.30 ± 0.50	0.988
HPI percentile, mean ± SD	0.66 ± 0.25	0.66 ± 0.26	0.978

Abbreviations: AVR: aortic valve replacement; HPI, Healthy Places Index; HMO, Health Maintenance Organization; sAS: severe aortic stenosis (inclusive of categorization of moderate to severe and worse aortic stenosis); SDoH, social determinants of health.

**Table 3:** Characteristics of patients who underwent transcatheter (TAVR) or surgical aortic valve replacement (SAVR) for severe aortic stenosis

	SAVR	TAVR	*p* value
N	96	488	
Age, mean ± SD	60.96 ± 11.18	79.63 ± 9.87	<0.001
Male, n (%)	78 (81.2)	318 (65.2)	0.003
Race, n (%)			0.249
White	64 (66.7)	375 (76.8)	
Asian	9 (9.4)	34 (7.0)	
Black	4 (4.2)	22 (4.5)	
Native Hawaiian or Pacific Islander	0 (0.0)	2 (0.4)	
American Indian or Alaska Native	1 (1.0)	2 (0.4)	
Other	15 (15.6)	39 (8.0)	
Unknown	3 (3.1)	14 (2.9)	
Ethnicity, n (%)			0.343
Non-Hispanic	79 (82.3)	428 (87.7)	
Hispanic	13 (13.5)	44 (9.0)	
Unknown	4 (4.2)	16 (3.3)	
Insurance, n (%)			<0.001
Private	46 (47.9)	251 (51.4)	
Medicare	37 (38.5)	181 (37.1)	
HMO	7 (7.3)	0 (0.0)	
Medicare Advantage	0 (0.0)	33 (6.8)	
Medicaid	3 (3.1)	8 (1.6)	
Unknown	3 (3.1)	15 (3.1)	
SDoH			
HPI, mean ± SD	0.17 ± 0.55	0.32 ± 0.46	0.048
HPI percentile, mean ± SD	0.59 ± 0.29	0.67 ± 0.24	0.045

Abbreviations: HMO, Health Maintenance Organization; HPI, Healthy Places Index; SDoH, social determinants of health.

Given the large number of patients missing SDoH data, we performed stratified analyses by SDoH availability to assess for associations between demographics, SDoH, and receipt of AVR for sAS (Table 4). In a multivariable logistic regression model including all patients regardless of SDoH availability, male sex (odds ratio 1.52, 95% confidence interval 1.09-2.11) and Medicare Advantage insurance coverage (compared to private insurance) (3.48, 1.18-14.92) were associated with greater odds of receiving AVR, whereas older age (0.96 per year, 0.95-0.98), traditional Medicare (0.48, 0.34-0.68), and Health Maintenance Organization insurance coverage (compared with private insurance) (0.24, 0.07-0.89) were associated with lower odds of receiving AVR (Table 4). Models were then repeated among patients with available SDoH data (n ​= ​454). This similarly identified a lower likelihood of receiving AVR among patients with advancing age (0.97 per year, 0.94-0.99), traditional Medicare (0.28, 0.17-0.45), and Health Maintenance Organization coverage (0.10, 0.01-0.67). In addition, compared with White patients, Black patients were 69% (0.31, 0.12-0.81) less likely to receive the treatment for AS, whereas patients of Hispanic ethnicity were 73% (0.27, 0.11-0.64) less likely to receive the treatment. There was not a significant difference in the treatment by HPI percentile (0.53, 0.19-1.42).

**Table 4:** Multivariable logistic regression comparing demographic factors and neighborhood level social determinant of health percentiles (Healthy Places Index [HPI]) among patients with severe aortic stenosis who did vs. did not undergo aortic valve replacement.

	All cohort (n ​= ​826)			With SDoH variable (n ​= ​454)		
	OR	95% CI	*p* value	OR	95% CI	*p* value
Age	0.96	(0.95, 0.98)	<0.001	0.97	(0.94, 0.99)	0.003
Gender						
Male	1.52	(1.09, 2.11)	0.012	1.34	(0.85, 2.11)	0.21
Race						
Asian	0.94	(0.52, 1.74)	0.84	1.55	(0.6, 4.56)	0.39
Black	0.57	(0.28, 1.16)	0.11	0.31	(0.12, 0.81)	0.016
Other	0.95	(0.56, 1.64)	0.85	1.44	(0.62, 3.63)	0.41
Unknown	0.68	(0.19, 2.38)	0.55	1.28	(0.3, 5.93)	0.74
Ethnicity						
Hispanic	0.62	(0.37, 1.06)	0.077	0.27	(0.11, 0.64)	0.004
Unknown	1.41	(0.4, 5.8)	0.61	1.66	(0.3, 13.73)	0.59
Insurance, n (%)						
Medicare	0.48	(0.34, 0.68)	<0.001	0.28	(0.17, 0.45)	<0.001
HMO	0.24	(0.07, 0.89)	0.024	0.1	(0.01, 0.67)	0.018
Medicare Advantage	3.48	(1.18, 14.92)	0.046	2.05	(0.62, 9.4)	0.29
Medicaid	0.66	(0.19, 3.11)	0.55	0.62	(0.06, 14.35)	0.71
Unknown	0.29	(0.13, 0.62)	0.001	0.2	(0.05, 0.8)	0.018
SDoH						
HPI percentile∗	-	-	-	0.53	(0.19, 1.42)	0.21

Abbreviations: HMO, Health Maintenance Organization; HPI: Healthy Places Index; OR, odds ratio; SDoH: neighborhood-level social determinants of health.

∗ Higher percentiles represent healthier neighborhood.

## Discussion

Although demonstrating the feasibility of designing and implementing a rules-based data engine to analyze over 30,000 echocardiograms, we identified over 2000 patients with some degree of untreated AS, including 242 with untreated sAS (11.2%), highlighting a concerning prevalence of undertreatment. We further observed that among these patients with untreated sAS, there was marked heterogeneity in the extent to which guideline-concordant treatment was eventually received and this heterogeneity was associated with variation in sociodemographic patient characteristics. Our results underscore the importance not only of accurately identifying and classifying severity of AS, but the potential of accessible systems-based algorithms that may be pragmatically implemented to facilitate guideline-based treatment and care.

As the national population continues to age, the rapid growth of patients suffering from AS is anticipated to further burden health care systems across the United States^1^ To effectively manage the impending increase in AS prevalence, health systems must have insights into current treatment and undertreatment patterns, as well as mechanisms to screen and bring these patients into care. The rules engine developed and evaluated in this study accomplishes both tasks. Specifically, it demonstrated excellent sensitivity and specificity for identifying sAS, with all misclassified cases identified as false positives. If implemented at scale, the reassuring lack of false negatives suggests that patients who need referral will not be missed. The rules engine is currently securely available in a Tableau dashboard to members of the cardiovascular team; however, we ultimately envision a role for this tool to be integrated into the EHR so that once patients undergo echocardiography that identifies severe AS, there is an automated process to coordinate their care and ensure referral for valve replacement. In addition, although this is currently being used at a single quaternary care center, the low-profile nature of the platform allows for ready expansion to additional institutions.

Underrecognition of sAS and associated delays in care have driven other groups to develop similar programs, however, with several key differences from our study.^9^^,^^10^ For example, EGNITE created a predictive “diagnostic precision algorithm,” that sought to capture sAS not identified in the original echocardiographic analysis.^9^ Conversely, Comprehensive Unified Regimen for Eliminating Valvular Heart Disease (CURE VHD) uses the developed clinical rules engine to identify patients in whom sAS is captured by echocardiography but are lost to follow-up or otherwise have their diagnoses unrecognized by their treating providers. This was demonstrated in the manual adjudication of our data, which was not performed in EGNITE, and demonstrated a high frequency of discordance between echocardiographic and clinical diagnosis of sAS.^9^ The MPIRIK study most closely mirrors our analysis; however, did not address the important and highly common scenario of discordance between quantitative and qualitative AS assessment.^10^ As such, CURE-VHD extends beyond these prior studies, using readily available data, without the need for expensive machine learning tools, to bring the large number of patients with recognized but untreated sAS into care.

Our study also revealed age-related disparities in receipt of AVR for sAS. As expected, we found the likelihood of undergoing valve replacement decreased with an increasing age. Prior studies have reported higher mortality rates among elderly patients undergoing either SAVR or TAVR, but it remains unclear whether this increased mortality contributes to treatment delays (out of hesitation for referral), results from treatment delays, or—most likely—a combination of both.^11^^,^^12^ These disparities may stem from age serving as a surrogate for severity of illness not captured in our data set, or age itself may contribute to undertreatment independent of comorbidities—suggesting a hesitancy to offer a potentially life prolonging treatment to older patients or for older patients to decline intervention. Among those who did receive treatment, patients undergoing SAVR were more likely to be younger than those undergoing TAVR, which is also in alignment with current clinical practice, although this may change as additional data become available on the use of TAVR at younger ages.^7^^,^^13^

Similar to prior studies, we also found persistent sex disparities in the sAS treatment. Compared to men, women are diagnosed with severe AS at older ages, are more often symptomatic at presentation, experience longer wait times, undergo fewer valve replacements, and consequently face higher 5-year mortality than men.^14^^,^^15^ Consistent with these findings, our study found women were significantly less likely to undergo AVR even after multivariable adjustment. Our results also align with prior literature showing women are less likely to undergo SAVR, as opposed to TAVR.^16^^,^^17^ These sex-related disparities reflect broader trends in cardiovascular care, including the underrecognition and undertreatment of acute coronary syndromes in women.^17^, ^18^, ^19^

Our findings also align with prior research demonstrating racial and ethnic disparities in the treatment of AS, with Black and Hispanic patients being less likely to undergo AVR.^20^, ^21^, ^22^ Interestingly, in our analysis this was most apparent among patients from whom SDoH data were available, although the trend persisted across the entire cohort and likely reflect broader social and economic factors that contribute to inequities in care.

We also identified treatment disparities based on insurance status, highlighting the impact of socioeconomic factors on access to care. In our national health care system, patients with private insurance—often those with greater financial means—are more likely to access cardiologists and receive timely referrals for valve replacements. Although we hope that care coordination pathways developed from this analysis can help reduce referral disparities, prior research shows that insurance status also affects postprocedural outcomes, with higher in-hospital mortality observed after valve replacement among patients with nonprivate insurance.^23^

Several limitations of this study merit consideration. First, the observational nature of the analyses focused on receiving treatment and modality of intervention precludes determination of causality, although this does not apply to the validation process of the rule engine. In addition, data were obtained from a single center, potentially limiting generalizability; for example, our sample’s insurance status included more private payors and less Medicare Advantage than the general population. Fortunately, the quantitative metrics used to classify AS severity are based on national guidelines and represent standard metrics reported in echocardiographic reports. We further narrowed the analysis to only transthoracic studies without prior valvular interventions to minimize error introduced by factors such as pressure recovery. As such, future studies will be required to evaluate the ability of similar rules-engines to grade prosthetic valve dysfunction. Sample size may also limit findings, particularly the sensitivity analysis. Future studies may focus on larger sample sizes, although this would likely require the use of currently clinically unavailable machine learning models. Finally, we could not fully control for all factors that influence decisions regarding candidacy for valvular intervention, particularly clinical comorbidities. As such, factors outside of this study may confound treatment decisions; however, given the alignment with prior findings, this effect is likely minimal.

## Conclusion

This study demonstrates the validity and capacity of a rules-engine to identify and classify the severity of AS in an unselected population. We found a high prevalence of untreated sAS particularly among women, older adults, and patients with nonprivate insurance coverage. These findings highlight the need for a systems-based approach to the identification, management, and treatment of AS to bring patients into care sooner and reduce disparities. Future efforts to expand this work to other valvular conditions may allow for a population-level approach to the management of valvular heart disease, offering the opportunity to address the growing morbidity and mortality of these conditions.

## Ethics Statement

Institutional Review Board approval was obtained from Cedars-Sinai Medical Center with a waiver for informed consent.

## Funding

This work was partially supported by grant funding from 10.13039/100006520Edwards LifeSciences.

## Disclosure Statement

Raj Makkar reports financial support was provided by Edwards Lifesciences Corporation; reports a relationship with Edwards Lifesciences Corporation that includes funding grants; reports a relationship with Boston Scientific Corporation that includes funding grants; reports a relationship with Abbott Vascular Inc that includes funding grants; reports a relationship with Medtronic Inc that includes funding grants; and reports a relationship with JenaValve Technology Inc that includes funding grants. Joseph Ebinger reports financial support was provided by Edwards Lifesciences Corporation.andreports a relationship with Edwards Lifesciences Corporation that includes consulting or advisory.

The other authors had no conflicts to declare.
