# Supplementary material for: Comprehensive Unified Regimen for Eliminating Undiagnosed/Untreated Aortic Valve Stenosis: Algorithm Validation for Identifying Aortic Stenosis and Treatment Disparities
Source: Struct Heart. 2025 Nov 5;10(2):100755. doi: 10.1016/j.shj.2025.100755 (PMC12810532; doi:10.1016/j.shj.2025.100755)
Supplement: Supplementary data [file mmc1.docx]

**Comprehensive Unified Regimen for Eliminating Undiagnosed/Untreated Aortic Valve Stenosis (CURE-AS)**

Supplemental Materials

Supplemental Table 1………………2

Supplemental Figure 1……………...3

**Correspondence.** Joseph Ebinger 127 S. San Vicente Blvd., Suite A3100 Los Angeles, CA 90048, USA, Phone (310) 423-2726**,** Email cda-research@cshs.org

**Supplemental Table 1.** Demographics of Patients with and without Neighborhood Level Social Determinants of Health (SDoH) Data.

|  | **With SDoH** | **Without SDoH** | **P-Value** |
| --- | --- | --- | --- |
| **N** | 454 | 372 |  |
| **Age, mean (SD)** | 79.08 (11.58) | 76.79 (13.13) | 0.008 |
| **Sex, n (%)** |  |  | 0.151 |
| Female | 176 (38.8) | 123 (33.1) |  |
| Male | 277 (61.0) | 249 (66.9) |  |
| Unknown | 1 (0.2) | 0 (0.0) |  |
| **Race, n (%)** |  |  | 0.009 |
| White | 351 (77.3) | 257 (69.1) |  |
| Asian | 31 (6.8) | 32 (8.6) |  |
| Black | 25 (5.5) | 17 (4.6) |  |
| Native Hawaiian or Pacific Islander | 2 (0.4) | 0 (0.0) |  |
| American Indian or Alaska Native | 0 (0.0) | 3 (0.8) |  |
| Other | 32 (7.0) | 50 (13.4) |  |
| Unknown | 13 (2.9) | 13 (3.5) |  |
| **Ethnicity, n (%)** |  |  | 0.001 |
| Non-Hispanic | 408 (89.9) | 300 (80.6) |  |
| Hispanic | 35 (7.7) | 56 (15.1) |  |
| Unknown | 11 (2.4) | 16 (4.3) |  |
| **Insurance, n (%)** |  |  | 0.001 |
| Private | 194 (42.7) | 179 (48.1) |  |
| Medicare | 214 (47.1) | 143 (38.4) |  |
| HMO | 5 (1.1) | 7 (1.9) |  |
| Medicare Advantage | 26 (5.7) | 10 (2.7) |  |
| Medicaid | 4 (0.9) | 10 (2.7) |  |
| Unknown | 11 (2.4) | 23 (6.2) |  |
| **Procedure, n (%)** |  |  |  |
| SAVR | 41 (9.0) | 55 (14.8) | 0.014 |
| TAVR | 282 (62.1) | 206 (55.4) | 0.059 |

**Supplemental Figure 1.** Multivariable regression on predictors of rules engine misclassification. LVEF: Left Ventricular Ejection Fraction

**
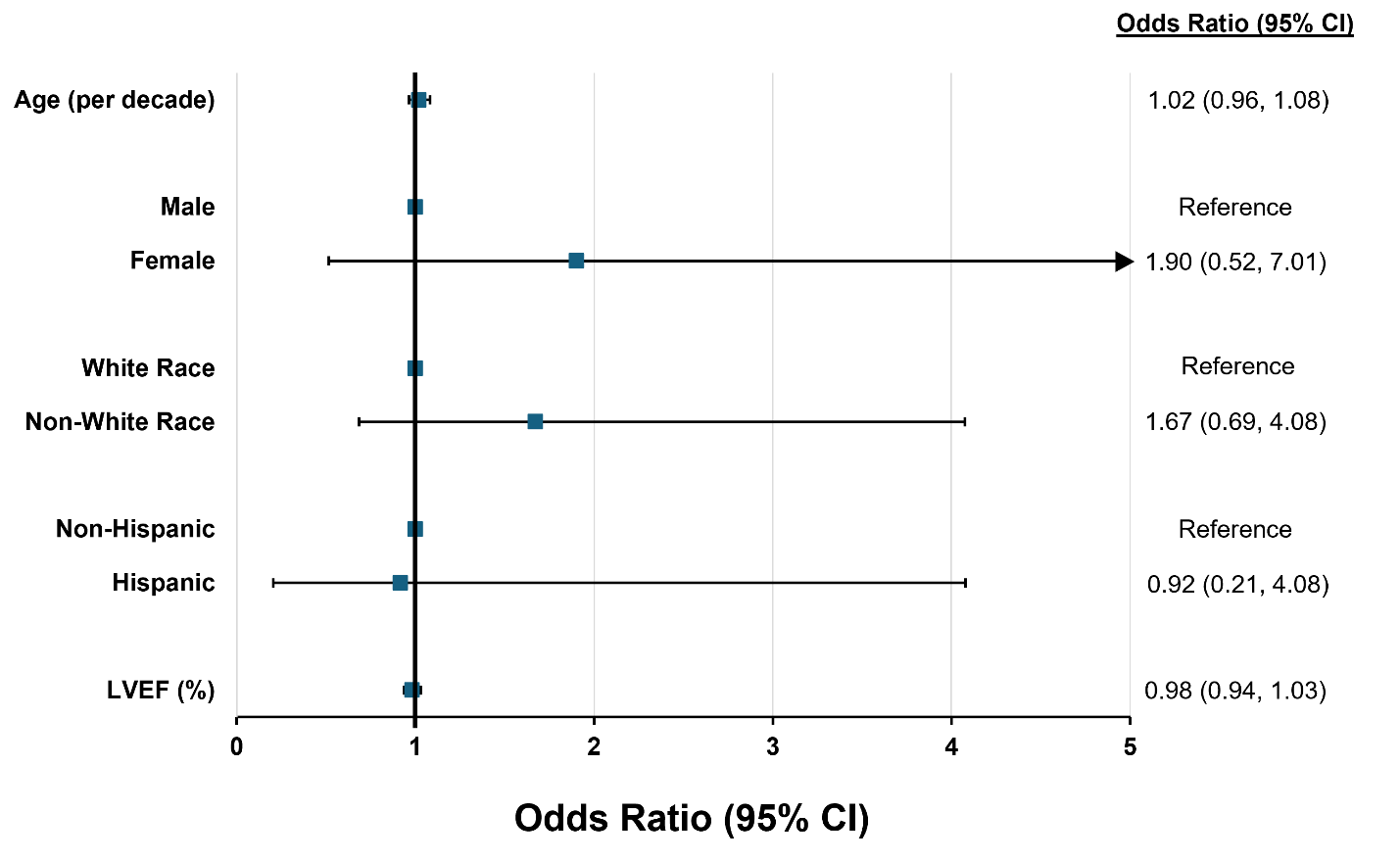
**
